# Glycogen synthase kinase-3β inhibition promotes lysosome-dependent degradation of c-FLIP_L_ in hepatocellular carcinoma

**DOI:** 10.1038/s41419-018-0309-3

**Published:** 2018-02-14

**Authors:** Na Zhang, Xiaojia Liu, Lu Liu, Zhesong Deng, Qingxuan Zeng, Weiqiang Pang, Yang Liu, Danqing Song, Hongbin Deng

**Affiliations:** 1 Institute of Medicinal Biotechnology, Chinese Academy of Medical Sciences & Peking Union Medical College, Beijing, 100050 China; 2 Medical College, Nanchang University, Nanchang, Jiangxi 330006 China

## Abstract

Glycogen synthase kinase-3β (GSK-3β) is a ubiquitously expressed serine/threonine kinase involved in a variety of functions ranging from the control of glycogen metabolism to transcriptional regulation. We recently demonstrated that GSK-3β inhibition triggered ASK1-JNK-dependent apoptosis in human hepatocellular carcinoma (HCC) cells. However, the comprehensive picture of downstream GSK-3β-regulated pathways/functions remains elusive. In this study, we showed that GSK-3β was aberrantly activated in HCC. Pharmacological inhibition and genetic depletion of GSK-3β suppressed the growth and induced caspase-dependent apoptosis in HCC cells. In addition, GSK-3β inhibition-induced apoptosis through downregulation of c-FLIP_L_ in HCC, which was caused by biogenesis of functional lysosomes and subsequently c-FLIP_L_ translocated to lysosome for degradation. This induction of the lysosome-dependent c-FLIP_L_ degradation was associated with nuclear translocation of transcription factor EB (TFEB), a master regulator of lysosomal biogenesis. Moreover, GSK-3β inhibition-induced TFEB translocation acts through activation of AMPK and subsequently suppression of mTOR activity. Thus our findings reveal a novel mechanism by which inhibition of GSK-3β promotes lysosome-dependent degradation of c-FLIP_L_. Our study shows that GSK-3β may become a promising therapeutic target for HCC.

## Introduction

Hepatocellular carcinoma (HCC) is one of the most common malignant tumors and the third leading cause of cancer-related death worldwide. More than 600,000 deaths are attributed to HCC each year^1^. The short-term prognosis of patients with HCC has improved recently due to advances in early diagnosis and treatment, but long-term prognosis remains unsatisfactory, as indicated by the low overall survival of 22–35% at 10 years after curative treatment^2,3^. Therefore, it is imperative to explore the oncogenic cellular signaling of which are implicated in the malignant phenotype of HCC^4^.

Glycogen synthase kinase-3 (GSK-3) is a ubiquitously expressed serine/threonine protein kinase that exists as two highly similar mammalian isoforms, GSK-3α, and GSK-3β^5^. GSK-3β is involved in myriad biologic functions and has emerged as a potential therapeutic target for treatment of various diseases including diabetes, Alzheimer’s disease, and affective disorders^6,7^. The roles of GSK-3β in cancer and tumor progression remain controversial^8,9^. Several studies suggested a possible role of GSK-3β as a tumor suppressor gene in HCC^10–12^, and consequently loss of GSK-3β expression and/or inhibition of its activity may contribute to HCC development. However, other studies have reported that inhibition of GSK-3β affect HCC cell survival and proliferation^13–15^, indicating that GSK-3β is a potential therapeutic target for this neoplasia. In line with this, we have shown that GSK-3β inhibition triggers apoptosis in HCC cells by mechanisms involving ASK1-JNK activation^16^, meanwhile others have observed GSK-3β inhibition reduced cell growth through Bax, TP53, and TGF-β signaling pathway^13,17^. Despite the general consensus supporting an important role for GSK-3β in the maintenance of HCC cell growth, a comprehensive picture of the underlying downstream GSK-3β effectors remains elusive.

Cellular FLICE-inhibitory protein (c-FLIP) is a death effector domain (DED)-containing family member that prevents induction of apoptosis mediated by death receptors (DR)^18^. Two isoforms of c-FLIP are commonly detected in human cells: a long form (c-FLIP_L_) and a short form (c-FLIP_S_). Both isoforms are recruited to the DISC, prevent procaspase-8 activation and block DR-mediated apoptosis, although through different mechanisms^19^. c-FLIP regulates life and death in various types of normal cells and tissues, such as lymphoid cells, and renders resistance to DR-mediated apoptosis in many types of cancer cells^20,21^. Dysregulation of c-FLIP expression has been shown to be associated with various diseases, such as cancer and autoimmune diseases, and c-FLIP might be a critical target for therapeutic intervention^22^. The levels of c-FLIP are regulated at both the transcriptional and post-translational levels. For example, miRNA-708 has been shown to regulate c-FLIP expression in HCC cells^23^. Meanwhile, it has been shown that c-FLIP expression is regulated through proteasome-dependent pathway in NSCLC cells^24,25^. Given that c-FLIP involved in a variety of cellular processes in different types of cells, it is of great interest to identify additional molecules or mechanisms responsible for the regulation of c-FLIP expression.

In this study, we further characterized the impact of GSK-3β in HCC cells rather than regulating ASK1-dependent apoptotic markers^16^. We identified GSK-3β inhibition suppressed the growth and induced apoptosis in HCC cells. In addition, GSK-3β inhibition was found to promote lysosome-dependent c-FLIP_L_ degradation, which was associated with elevated nuclear localization of transcription factor EB (TFEB). Our study thus identified a previously undiscovered mechanism for regulation of c-FLIP_L_ expression and provides a novel therapeutic strategy for modulating lysosomal function in HCC.

## Results

### GSK-3β is expressed and active in HCC

**Fig. 1:**
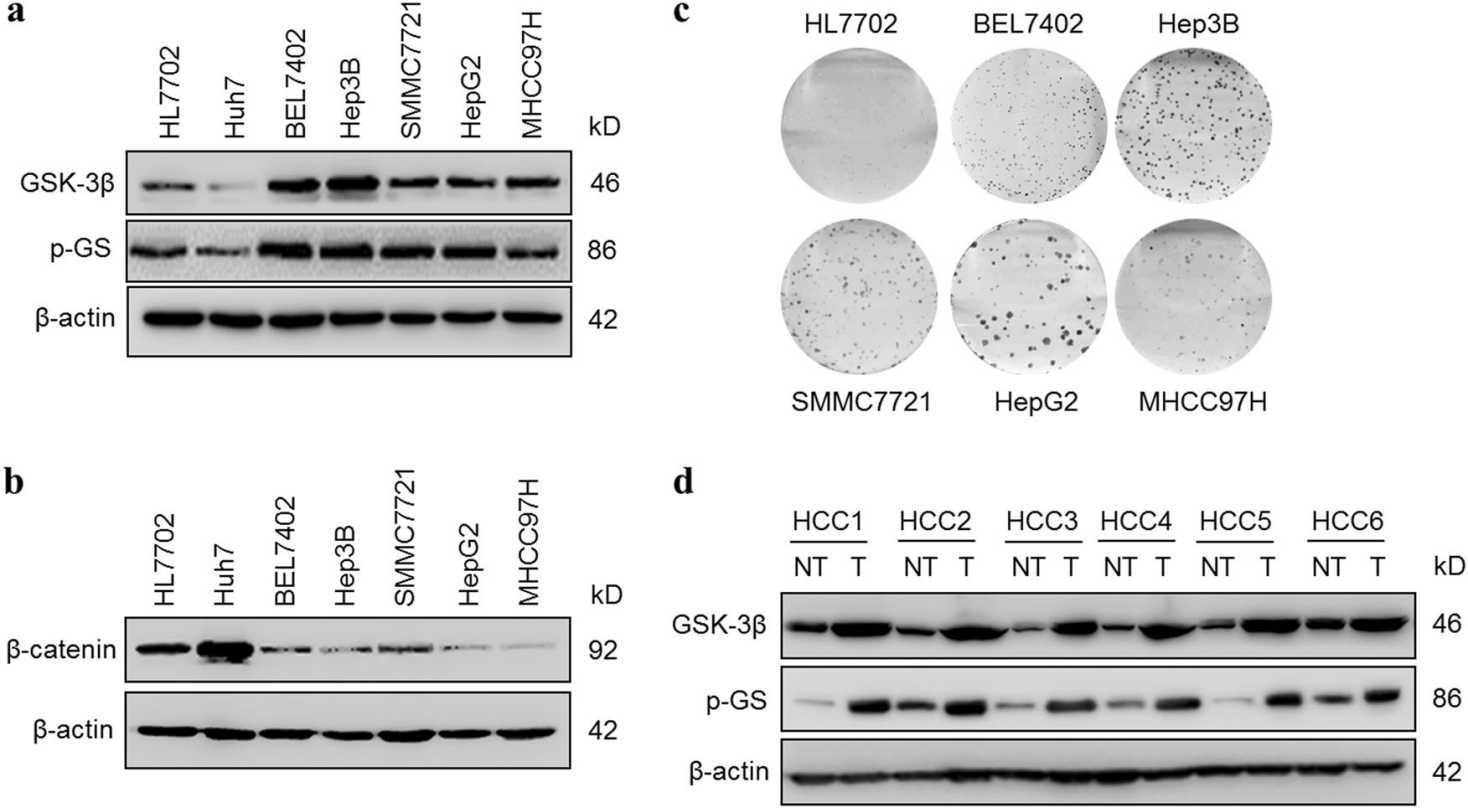
GSK-3β is expressed and active in HCC cells. **a** The whole-cell protein lysates from six different human HCC cell lines (Huh7, BEL7402, Hep3B, SMMC7721, HepG2, MHCC97H) and one normal hepatocyte line HL7702 were analyzed by immunoblotting (IB) with antibodies against GSK-3β and phospho-GS (p-GS), β-actin was used as the internal control. **b** The β-catenin levels in whole-cell protein lysates of the seven indicated cell lines were determined by IB. **c** HL7702, BEL7402, Hep3B, SMMC7721, HepG2, and MHCC97H cells were planted in 6-well plates, the formed clones was showed by hematoxylin dye after 15 days. **d** Expressions of GSK-3β and p-GS were analyzed by IB in HCC tumor tissues (T) and paired non-tumorous tissues (NT)

To determine the role of GSK-3β in HCC development, we first examined its expression levels in six HCC cell lines and one normal hepatocyte line HL7702. Immunoblotting (IB) results revealed that the five (BEL7402, Hep3B, SMMC7721, HepG2, and MHCC97H) of the six human HCC cell lines demonstrated elevated levels of GSK-3β expression, as compared with the normal line HL7702, albeit to varied extent (Fig. 1a, upper panel). In addition, all the cell lines with elevated GSK-3β expression showed higher levels of phosphorylation of glycogen synthase (p-GS), a primary GSK-3β substrate, as compared with normal HL7702, suggesting that GSK-3β is active in HCC cells (Fig. 1a, middle panel). To further assess the activity of GSK-3β, we measured it’s another substrate of β-catenin^26^. Consistent with the high GSK-3β activity in HCC cells, we detected low β-catenin protein levels in BEL7402, Hep3B, SMMC7721, HepG2, and MHCC97H cells (Fig. 1b). Moreover, the active GSK-3β was also related to the tumorigenicity of HCC cell lines as determined by colony formation (Fig. 1c). The cells with higher levels of GSK-3β in HCC cells formed more colonies than that in normal HL7702 (Supplementary Figure S1). To gain a better understanding of the role of GSK-3β in HCC, we tried to determine the expression level of GSK-3β using clinical specimens of HCC. IB analysis revealed that increased protein expression level of GSK-3β and p-GS in tumor tissues compared with their normal counterparts (Fig. 1d). These data indicate that high levels of GSK-3β expression and activity are the features of HCC.

### Pharmacological inhibition and genetic depletion of GSK-3β inhibits the proliferation/survival and induces apoptosis in HCC cells

**Fig. 2:**
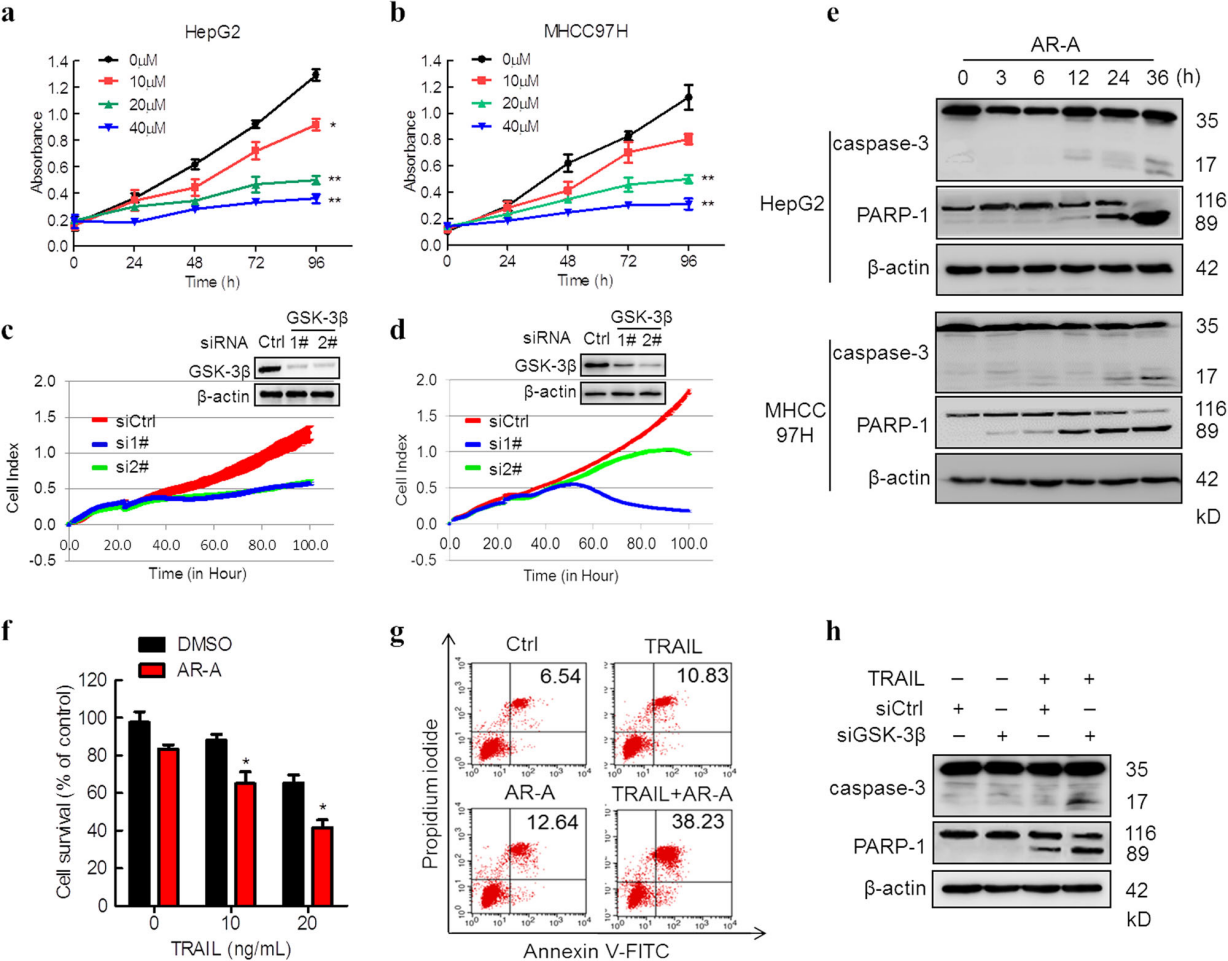
Inhibition of GSK-3β suppresses the growth and induces caspase-dependent apoptosis in HCC cells. **a**, **b** HepG2 and MHCC97H cells were treated with AR-A at the indicated concentrations, cell viability was measured by MTS assay. Data are presented as means ± S.D. (*n* = 3). ^*^P < 0.05, ^**^P < 0.01 compared with DMSO group. **c**, **d** HepG2 and MHCC97H cells were transfected with siRNAs targeting GSK-3β and the scramble control, cell index was monitored by cell impedance assay. Data was analyzed using the RTCA Software 1.2 program (Roche Diagnostics). All data is presented as the mean (*n* = 2) normalized cellular index over time. **e** HepG2 and MHCC97H cells were treated with 20 μM AR-A for the indicated time. Caspase-3 and PARP-1 cleavage were determined by IB, β-actin was used as the internal control. **f** MHCC97H cells were treated with 20 μM AR-A alone, different concentrations of TRAIL alone, or their respective combinations. After 24 h, the cell viability was determined by MTS assay. (*n* = 3). ^**^P < 0.01 compared with DMSO group. **g** MHCC97H cells were treated with 10 ng/mL TRAIL alone, 20 μM AR-A alone, and their respective combination as indicated. After 24 h, the apoptosis was measured by Annexin V/PI staining. **h** MHCC97H cells were transfected with GSK-3β or control siRNAs for 36 h, followed by 50 ng/mL TRAIL treatments for 8 h, the cleavage of caspase-3 and PARP-1 were monitored by IB

To address whether GSK-3β indeed affects the cell survival of HCC cells, we analyzed the effects of pharmacological inhibition and genetic depletion of GSK-3β in HepG2 and MHCC97H cells because these cell models showed moderate GSK-3β expression (Fig. 1a). Indeed, we observed a significantly decreased in cell proliferation/survival of HepG2 and MHCC97H cells, upon treatment with the specific GSK-3β inhibitor AR-A014418 (AR-A, Supplementary Figure S2a) in both dose-dependent and time-dependent manners as determined by MTS and LDH release assays (Fig. 2a, b, Supplementary Figure S2b). To exclude potential off-target effects of the inhibitor for cell proliferation/survival, HepG2 and MHCC97H cells were transfected with two different siRNAs targeting GSK-3β, and cell index was monitored by impendence assay. Significant reduction in the cell index of HepG2 and MHCC97H cells was observed following two GSK-3β RNA interference as compared with non-targeting control (Fig. 2c, d).

To further explore the effects of GSK-3β inhibition on HCC cell function, we sought to determine whether inhibition of GSK-3β would induce apoptosis in HCC cells. AR-A treatment induced the cleavage of caspase-3 and PARP-1 in HepG2 and MHCC97H cells, which are the critical markers of apoptosis (Fig. 2e). Furthermore, the apoptosis of HCC cells induced by AR-A was inhibited by Z-VAD-FMK, a pancaspase inhibitor (Supplementary Figure S3a), indicating that caspase-mediated apoptosis is involved in AR-A-induced decrease in proliferation/survival of HCC cells. Previous work has shown that GSK-3β inhibition enhances TRAIL-induced apoptosis in NSCLC cells^24^. Therefore, we determined whether inhibition of GSK-3β also augmented TRAIL-induced apoptosis in HCC cells. Indeed, the combination of TRAIL with AR-A exerted much more potent effects than TRAIL or the inhibitors alone in decreasing the survival of MHCC97H cells (Fig. 2f). In agreement, the combinations were also more potent than each single agent alone in inducing apoptotic cell death as measured by Annexin V-FITC/PI assay (Fig. 2g). In addition, Hoechst 33342 staining assays demonstrated the apoptotic characteristics in HepG2 cells treated with AR-A or combination of TRAIL (Supplementary Figure S3b). Furthermore, necrostatin-1, a specific inhibitor of necroptosis, had no effect on AR-A or AR-A/TRAIL-induced decrease in survival of HCC cells (Supplementary Figure S3c), indicating that apoptosis, but not necroptosis, is the major process involved in AR-A-induced decrease in proliferation/survival of HCC cells. Moreover, consistent with these results, the combination of GSK-3β siRNA and TRAIL was much more potent in inducing cleavage of caspase-3 and PARP-1 (Fig. 2h). Together, these results suggest that GSK-3β inhibition suppresses the proliferation/survival and induces caspase-dependent apoptosis in HCC cells.

### GSK-3β inhibition downregulates the expression of c-FLIP_L_ in HCC

**Fig. 3:**
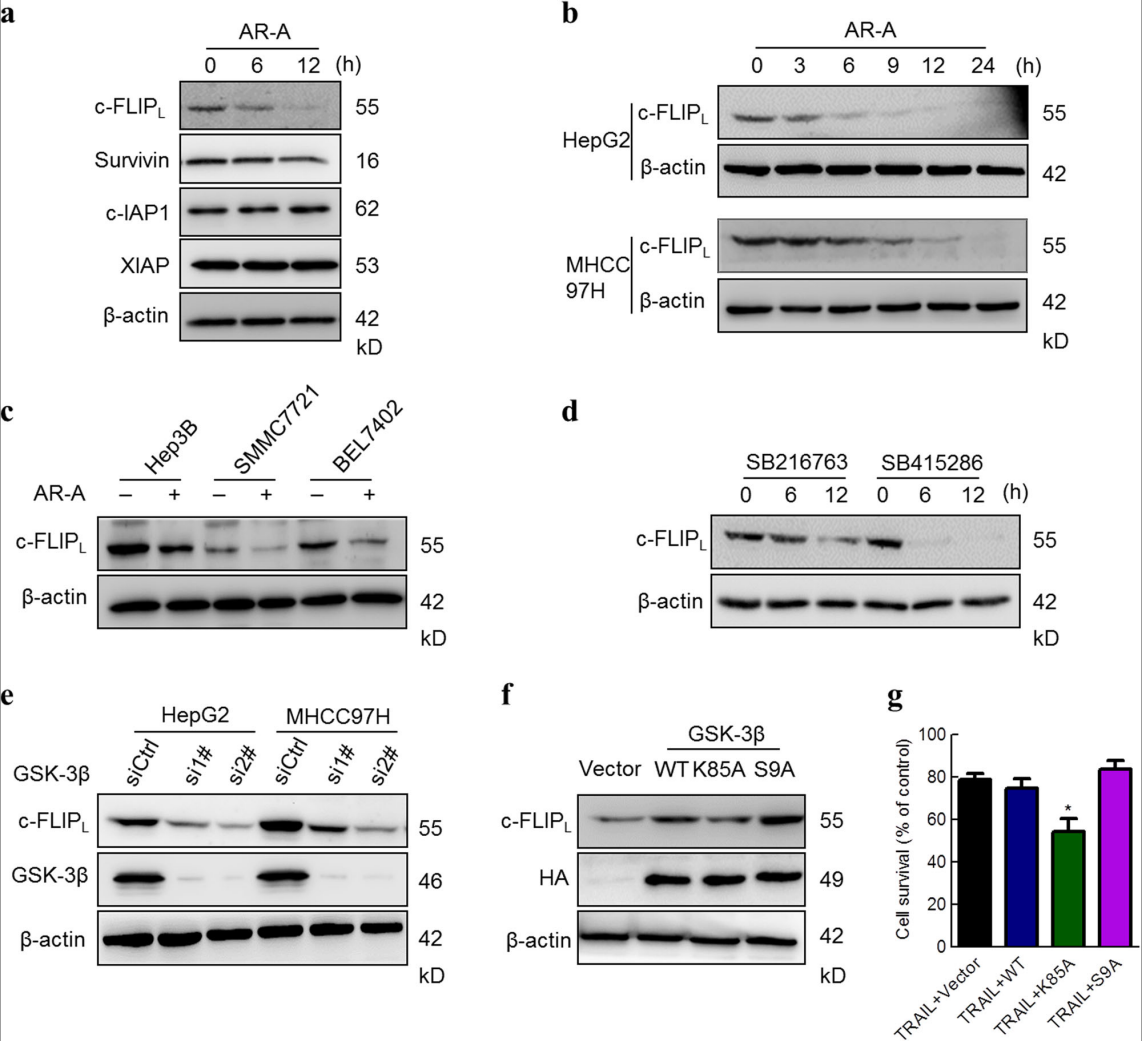
Modulation of GSK-3β activity alters c-FLIP_L_ levels in HCC. **a** MHCC97H cells were treated with 20 μM AR-A for the indicated time points, the expression levels of c-FLIP_L_, Survivin, c-IAP1, and XIAP were determined by IB. **b** HepG2 and MHCC97H cells were treated with AR-A for the indicated time, the c-FLIP_L_ levels was measured by IB. **c** Three HCC cell lines (Hep3B, SMMC7721, and BEL7402) were treated with AR-A for 6 h. The protein level of c-FLIP_L_ was measured by IB. **d** HepG2 cells were treated with the specific GSK-3β inhibitors SB216763 (20 μM) and SB415286 (20 μM) for the indicated time, the protein level of c-FLIP_L_ was measured by IB. **e** HepG2 and MHCC97H cells were transfected with siRNAs targeting GSK-3β and the scramble control for 48 h, and then the whole-cell protein lysates was probed with the antibodies against c-FLIP_L_, GSK-3β and β-actin. **f**, **g** HepG2 cells were transfected with empty vector or expression plasmids carrying WT, K85A or S9A of GSK-3β for 48 h, the expression levels of c-FLIP_L_ and HA-GSK-3β were measured by IB (**f**), the cell viability was determined by MTS assay after cells were treated with 10 ng/mL TRAIL for 24 h (**g**). (*n* = 3). ^*^P < 0.05 compared with TRAIL + vector group

To clarify the possible contribution of other factor(s) to GSK-3β inhibition-induced, caspase-dependent apoptotic cell death, we examined whether GSK-3β modulates the expression of cellular caspase antagonists. Among the tested inhibitor of apoptosis proteins (IAPs), c-IAP1 and XIAP protein levels were not altered by treatment with AR-A, although survivin protein level was slightly reduced (Fig. 3a, middle panel). Interestingly, the protein levels of c-FLIP_L_, a caspase-8 inhibitor, were strikingly reduced after treating with AR-A (Fig. 3a, upper panel). The changes of c-FLIP_S_ levels were not detected in these cells in the absence or presence of AR-A. In addition, the levels of c-FLIP_L_ were reduced quickly after incubation with AR-A for 6 h in HepG2 and MHCC97H cells (Fig. 3b), indicating that inhibition of GSK-3β activity affect c-FLIP_L_ level in HCC cells. This was further confirmed by AR-A treatment reducing the levels of c-FLIP_L_ in other HCC cells including Hep3B, SMMC7721, and BEL7402 (Fig. 3c). Next, we examined the effects of other GSK-3β inhibitors on c-FLIP_L_ expression and found that both SB216763 and SB415286 decreased the levels of c-FLIP_L_ (Fig. 3d) in HepG2 cells.

Moreover, we further inhibited GSK-3β by knocking down its expression using siRNAs and determined their impact on c-FLIP_L_ levels. As shown in Fig. 3e, silencing of GSK-3β by two different siRNAs greatly decreased the levels of c-FLIP_L_ in HepG2 and MHCC97H cells. Alternatively, we enforced expression of the wild type GSK-3β (HA-GSK-3β WT), the kinase dead type (HA-GSK-3β K85A), and the constitutively active type (HA-GSK-3β S9A) in HepG2 cells and then examined their impact on c-FLIP_L_ levels. As presented in Fig. 3f, overexpressing HA-GSK-3β WT increased the level of c-FLIP_L_, albeit less effectively than HA-GSK-3β S9A. In contrast, overexpression of HA-GSK-3β K85A reversed the GSK-3β-induced increase in c-FLIP_L_ (Fig. 3f). Furthermore, the combination of TRAIL with GSK-3β K85A exerted much more potent effects than GSK-3β WT or S9A in decreasing the survival of HCC cells (Fig. 3g). Additionally, overexpressing myc-c-FLIP_L_ decreased AR-A-induced apoptosis in HepG2 cells (Supplementary Figure S4). Taken together, these results clearly indicated that GSK-3β inhibition downregulates c-FLIP_L_ levels in HCC cells.

### Lysosomal pathway contributes to GSK-3β inhibition-mediated downregulation of c-FLIP_L_

**Fig. 4:**
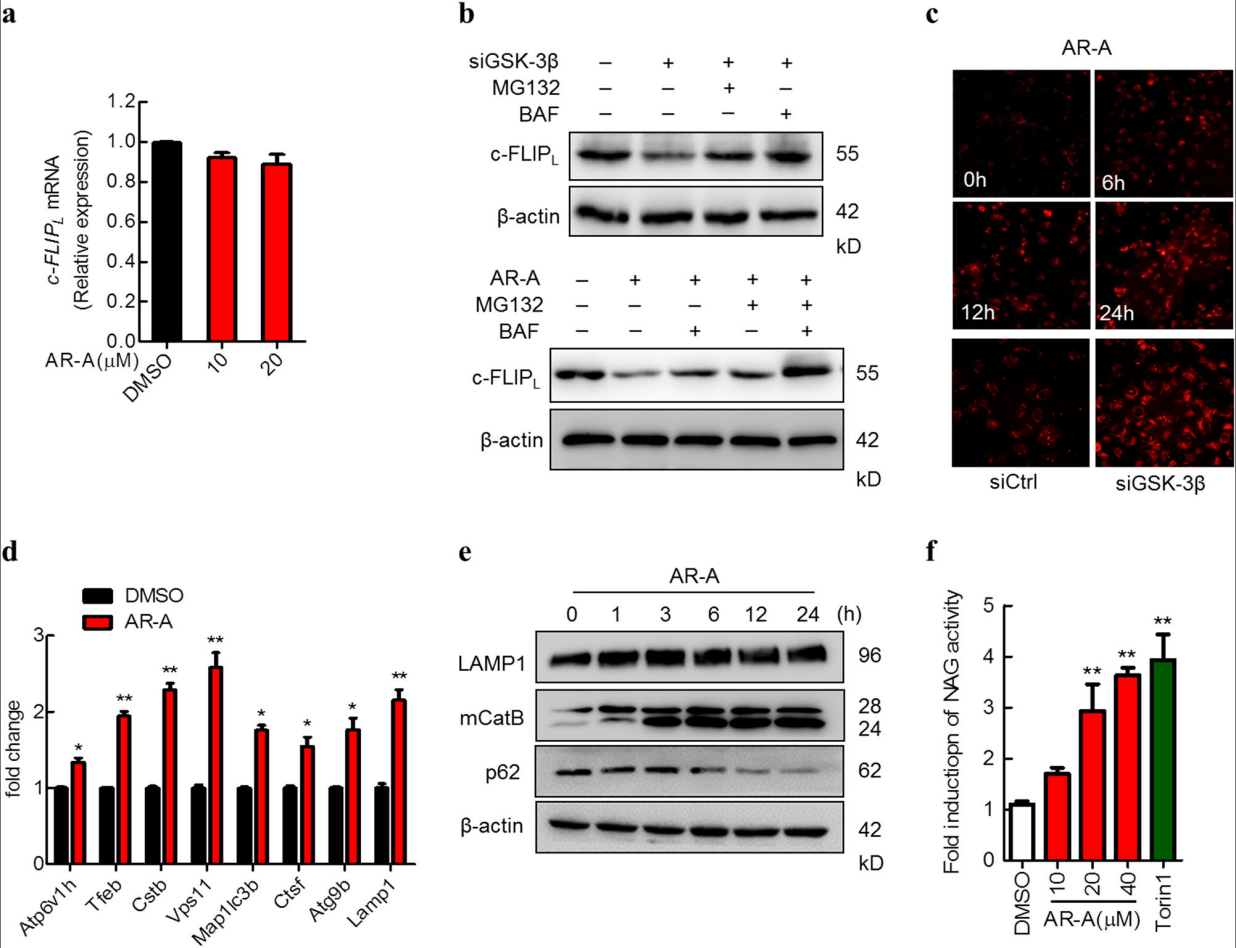
Lysosome pathway contributes to GSK-3β inhibition-mediated c-FLIP degradation. **a** HepG2 cells were treated with indicated concentrations of AR-A for 12 h. The mRNA level of c-FLIP_L_ was measured by qRT-PCR. **b** HepG2 cells were transfected with siRNAs targeting GSK-3β, followed by the autophagy inhibitor BAF (25 nM) or the proteasome inhibitor MG132 (10 µM) treatment for 12 h; or pretreated with MG132 (10 µM), BAF (25 nM) for 12 h, followed by AR-A treatment for 6 h. The protein level of c-FLIP_L_ was detected by IB. **c** MHCC97H cells were treated with 20 μM AR-A for the indicated time points, or transfected with GSK-3β siRNA for 48 h, followed by staining with the lysosome probe of LysoTracker Red. The red fluorescence images were monitored by microscope (20× scale). **d** MHCC97H cells were treated with 20 μM AR-A for 6 h and subjected to qRT-PCR analysis. (*n* = 3). ^*^P < 0.05, ^**^P < 0.01 compared with DMSO group. **e** MHCC97H cells were treated with 20 μM AR-A for the indicated time points, the levels of lysosome related proteins including LAMP1, mature-cathepsin B (mCatB), and p62 were determined by IB. **f** Relative lysosomal NAG activity of AR-A and Torin1-treated MHCC97H cells. (*n* = 3). ^**^P < 0.01 compared with DMSO group

Protein degradation is one of the main strategies involved in turning off protein functions in biological processes. At least two systems exist for protein degradation, including the ubiquitin-proteasome and lysosomal pathways^27,28^. It has been suggested that the ubiquitin-proteasome pathway contributed to c-FLIP_L_ degradation^25,29^, we examined whether other pathway involved in GSK-3β inhibition-mediated c-FLIP_L_ degradation. Before these experiments, we determined if inhibition of GSK-3β affects c-FLIP_L_ at the mRNA level. Using qRT-PCR, we did not detect any changes in c-FLIP_L_ mRNA levels in cells exposed to AR-A (Fig. 4a), indicating that GSK-3β inhibition-induced c-FLIP_L_ reduction does not occur at the transcriptional level. On the other hand, GSK-3β knockdown-mediated degradation of c-FLIP_L_ was inhibited by the proteasome inhibitor MG132 and lysosomal inhibitor Bafilomycin (BAF) (Fig. 4b, upper panel). Moreover, when AR-A co-treated HepG2 cells with MG132 or BAF, c-FLIP_L_ degradation was partially restored by BAF or MG132 alone but completely restored by a combination of BAF and MG132 (Fig. 4b, lower panel), indicating that GSK-3β inhibition-mediated c-FLIP_L_ degradation is mediated through both the proteasome-dependent and lysosome-dependent pathway. Therefore, it is plausible to speculate that the lysosome function was regulated by GSK-3β, resulted in the accelerated degradation of c-FLIP_L_. To confirm lysosomal function indeed affected by GSK-3β, we next examined the change in lysosome numbers using LysoTracker Red. Interestingly, inhibition of GSK-3β activity by AR-A induced a time-dependent increase in LysoTracker Red staining, similar to that caused by knocking down GSK-3β by siRNA (Fig. 4c).

To further confirm our findings, we investigated the changes of lysosomal-associated genes and proteins in the presence of GSK-3β inhibitor. In agreement with the LysoTracker Red staining results, we observed that AR-A upregulated many lysosome-related genes including *lamp1*, *ctsb*, *tfeb*, etc. (Fig. 4d). Meanwhile, AR-A increased the levels of LAMP1 (a lysosomal membrane protein marker) and Cathepsin B (CatB, a lysosomal protease), which indicated the number of lysosomes increased. In addition, the level of p62, one ubiquintation substrate degraded in lysosome, becoming lower after GSK-3β inhibition (Fig. 4e). Furthermore, AR-A increased lysosomal protease activities in MHCC97H cells, as measured by β-N-acetylglucosaminidase (NAG) assays (Fig. 4f). Taken together, these data suggest that inhibition of GSK-3β induce biogenesis of functional lysosomes, thus promote the degradation of c-FLIP_L_.

### Inhibition of GSK-3β promotes translocation of c-FLIP_L_ to lysosomes

**Fig. 5:**
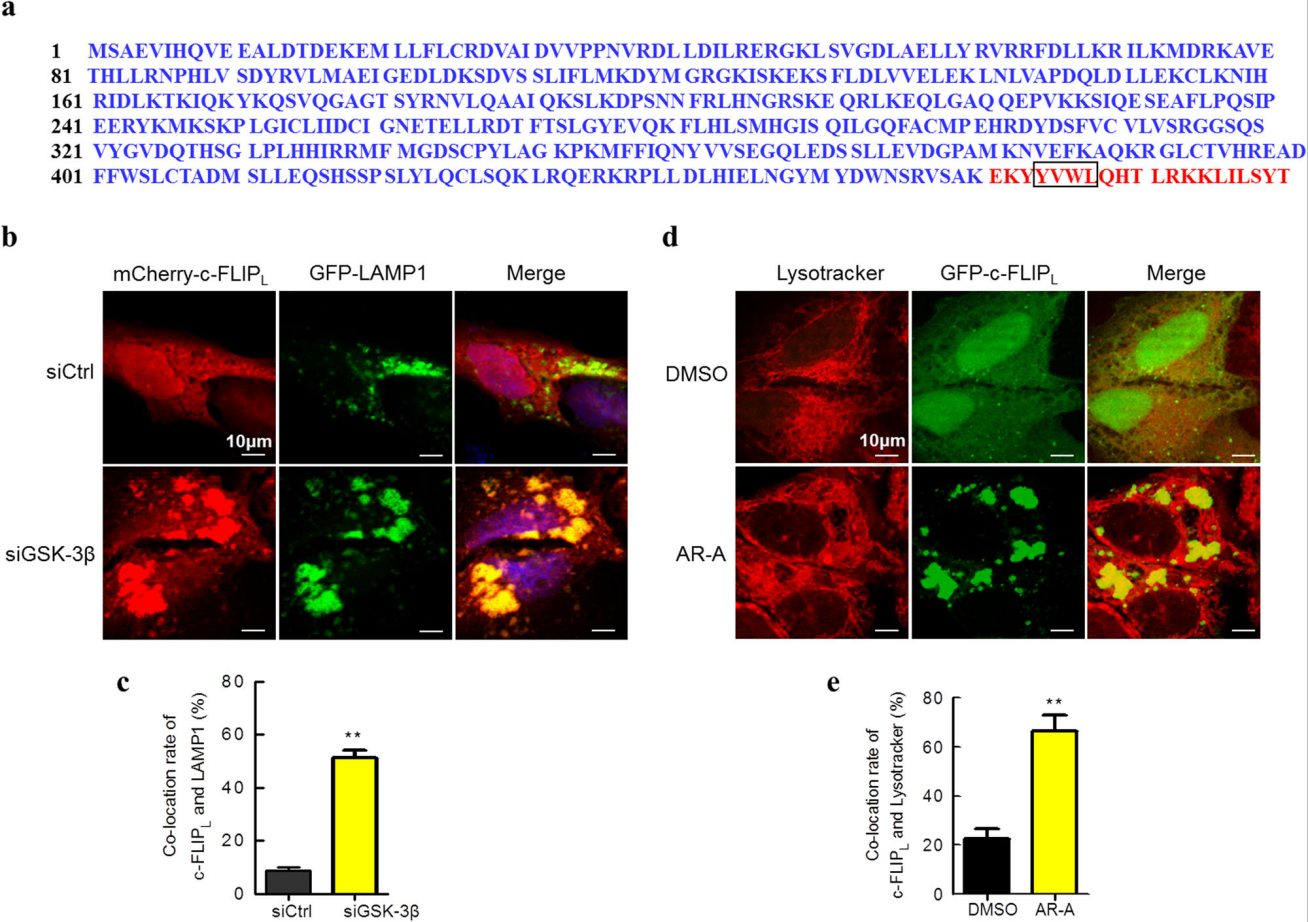
Inhibition of GSK-3β promotes translocation of c-FLIP_L_ to lysosomes. **a** Bioinformatic analysis of the c-FLIP_L_ amino acid sequence. c-FLIP_L_ contains a signal peptide (aa 461-480, red) including a tyrosine-based motif YVWL associated with lysosomes. **b** HepG2 cells were transfected with siRNAs targeting GSK-3β and the scramble control. After 24 h, cells were exogenously expressed with mCherry-c-FLIP_L_ and GFP-LAMP1 plasmids. The subcellular distribution of c-FLIP_L_ and LAMP1 were analyzed by confocal microscope. **c** The quantification of c-FLIP_L_ co-localization with LAMP1. (*n* = 3). ^**^P < 0.01 compared with siCtrl group. **d** MHCC97H cells were transfected with GFP-c-FLIP_L_. After 48 h, cells were treated with 20 μM AR-A or DMSO for 8 h, followed by Lysotracker Red staining, the subcellular distribution of c-FLIP_L_ and LysoTracker Red were analyzed by confocal microscope. **e** The quantification of c-FLIP_L_ co-localization with Lysotracker Red. (*n* = 3). ^**^P < 0.01 compared with DMSO group

We conducted a bioinformatic analysis of the c-FLIP_L_ amino acid sequence to identify consensus sequences indicative of its subcellular compartment localization (http://www.uniprot.org/uniprot/ O15519 and Fig. 5a). A signal peptide including a tyrosine based motif YVWL (Y, tyrosine; V, valine; W, tryptophan; and L, leucine) was found at amino acid position 464. This peptide belongs to the family of peptide motifs with the general YXXu structure (where Y represents tyrosine, X any amino acid, and u a bulky hydrophobic residue such as leucine). Such motifs are described in various proteins associated with lysosomes, such as CD63, LAMP-1, LAMP-2, or CTLA-4^30^.

We thus hypothesized that the intracellular fraction of c-FLIP_L_ could be located in the lysosomal compartment. Confocal microscopy analysis indicated that a fraction of mCherry-c-FLIP_L_ did localize in lysosomes in control HepG2 cells, and an increased amount of mCherry-c-FLIP_L_ was found to localize in lysosomes when treatment with GSK-3β siRNA (Fig. 5b, c), indicating that knocking down of GSK-3β promoted translocation of c-FLIP_L_ to the lysosomes. In addition, AR-A treatment induced colocalization of GFP-c-FLIP_L_ with the LysoTracker Red in MHCC97H cells (Fig. 5d, e). These observations together suggest that inhibition of GSK-3β promotes translocation of c-FLIP_L_ to lysosomes for proteolysis.

### GSK-3β inhibition induces TFEB translocation for c-FLIP_L_ degradation

**Fig. 6:**
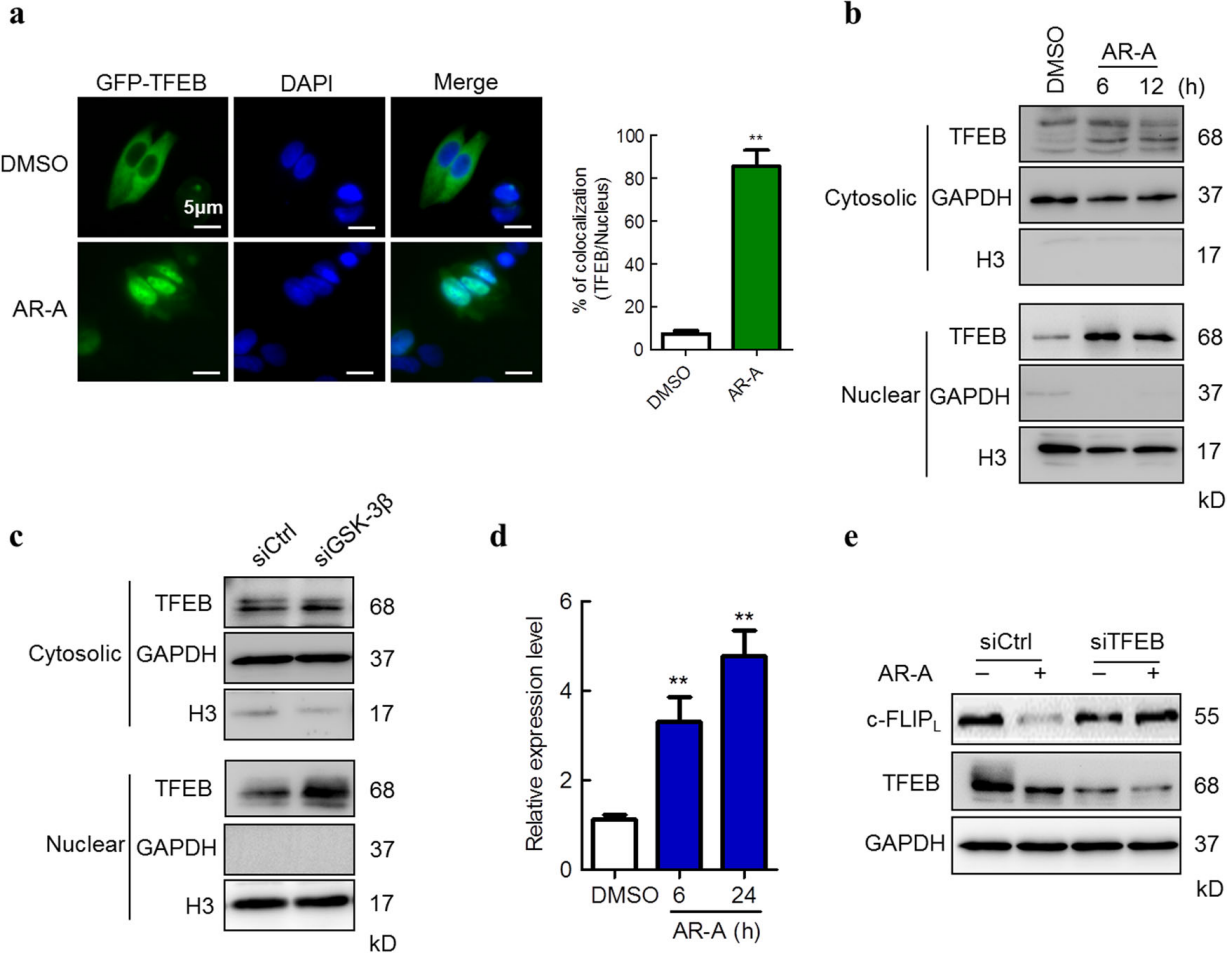
GSK-3β inhibition promotes TFEB nuclear localization. **a** HepG2 cells were transfected with GFP-TFEB plasmid, and then incubated with DMSO or AR-A (20 μM) for 6 h. The subcellular distribution of TFEB was analyzed by microscopy. Nuclear was stained with DAPI (scale bars, 5 µm). The right panel was the percentage of TFEB co-location with DAPI. (*n* = 3). ^**^P < 0.01 compared with DMSO-treated cells. **b** HepG2 cells were incubated with DMSO or AR-A (20 μM) for the indicated time. Cytosolic and nuclear proteins were fractionated and subjected to IB analyses of TFEB subcellular location. **c** HepG2 cells were transiently transfected siRNAs targeting GSK-3β and the scramble control. Forty-eight hour later, cytosolic and nuclear proteins were fractionated and subjected to IB analyses. **d** HepG2 cells were transfected with a pGL2-2 × CLEAR vector and the pRL-TK as an internal control for 24 h, followed by 20 μM AR-A treatment for 6 or 24 h. The level of luciferase activities were determined as described. (*n* = 3). ^**^P < 0.01 compared with DMSO-treated cells. **e** HepG2 cells were transfected with siRNAs targeting TFEB and the scramble control for 48 h, followed by DMSO or AR-A (20 μM) treatment for 6 h, the levels of c-FLIP_L_ and TFEB were measured by IB

Lysosome biogenesis can be triggered by the transcription factors TFEB, which increasing the number of lysosomes and promoting protein degradation^31,32^. Having demonstrated that inhibition of GSK-3β induces biogenesis of lysosomes (Fig. 4), we next sought to investigate the role of GSK-3β in regulating TFEB activity in HCC cells. To this end, we incubated HepG2 cells transfected with GFP-TFEB with AR-A. Fluorescence of TFEB showed that GSK-3β inhibition resulted in efficient TFEB nuclear translocation (Fig. 6a). In addition, IB analysis of TFEB in nuclear and cytosol fractionations revealed that AR-A treatment induced an increase of TFEB in the nuclear fraction (Fig. 6b). Same results were obtained when we examined the TFEB nuclear translocation after GSK-3β was knocked down by siRNA (Fig. 6c).

TFEB transcriptionally regulated a gene network termed CLEAR (coordinated lysosomal expression and regulation), which is the master regulator for lysosomal biogenesis. Subsequently, we found that the CLEAR luciferase activity was dramatically increased by AR-A treatment in HepG2 cells (Fig. 6d). To evaluate the relevance of TFEB on c-FLIP_L_ degradation, we knocked down TFEB expression by siRNA in HepG2 cells. Compared to control cells, silencing of TFEB attenuated the reduction in c-FLIP_L_ expression induced by GSK-3β inhibition (Fig. 6e).Taken together, these data suggest that TFEB nuclear translocation is essential to mediate the effects of GSK-3β inhibition on c-FLIP_L_ degradation.

### AMPK-mTOR signaling contributes to GSK-3β inhibition-induced TFEB nuclear translocation

**Fig. 7:**
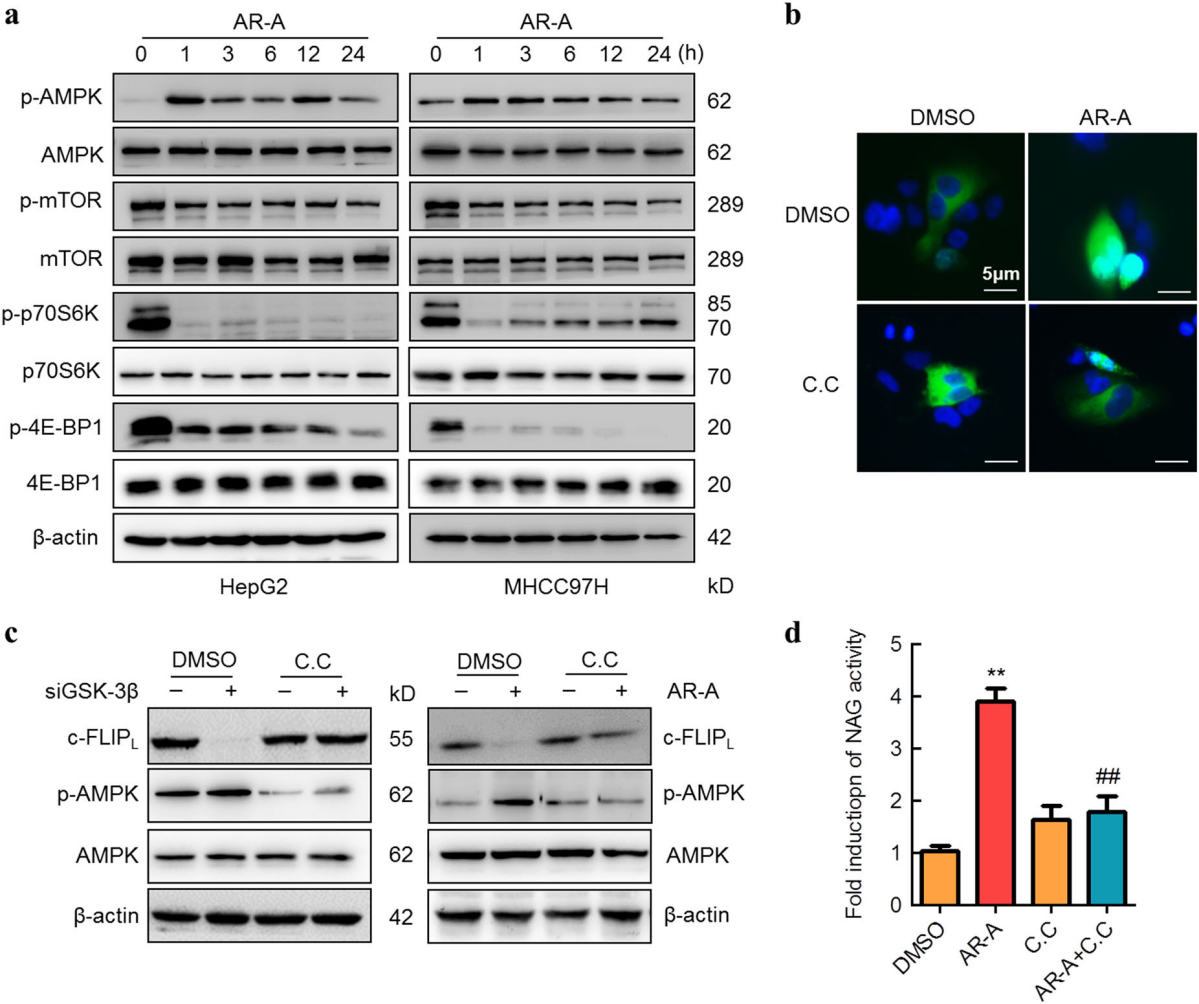
AMPK-mTOR pathway contributes to GSK-3β inhibition-induced TFEB nucleus translocation. **a** HepG2 and MHCC97H cells were treated with AR-A for the indicated time points, the levels of p-AMPK, AMPK, p-mTOR, mTOR, p-p70S6K, p70S6K, p-4E-BP1, and 4E-BP1 were determined by IB. **b** HepG2 cells were transfected with GFP-TFEB plasmid, followed by pretreatment with or without Compound C (C.C) for 2 h and AR-A incubated for 6 h. The subcellular distribution of TFEB was analyzed by microscopy. Nuclear was stained with DAPI (scale bars, 5 µm). **c** MHCC97H cells were transfected with siRNAs targeting GSK-3β for 48 h and treated with or without C.C for 12 h; or pretreated with DMSO or C.C for 2 h, then followed by AR-A treatment for 12 h. The expression levels of c-FLIP_L_, p-AMPK, and AMPK were measured by IB. **d** MHCC97H cells were pretreated with C.C for 2 h, followed by AR-A treatment for 12 h. The lysosomal NAG activity was measured by NAG assay. (*n* = 3). ^**^P < 0.01 compared with DMSO-treated cells, ^##^*P* < 0.01 compared with AR-A-treated cells

Recent studies have shown that inhibition of GSK-3β activity resulted in a significant increase of AMP-activated serine/threonine protein kinase (AMPK) activity^33,34^. Since TFEB activity is regulated by AMPK and mammalian target of rapamycin (mTOR)^35,36^. We examined the potential roles of AMPK-mTOR pathway in modulating GSK-3β-mediated TFEB nuclear translocation. As expected, our results demonstrated an increase of AMPK phosphorylation at Thr172 in a time-dependent manner following treatment with AR-A in both HepG2 and MHCC97H cells (Fig. 7a, upper panel). The increase of AMPK phosphorylation was accompanied by a reduction in phosphorylated mTOR, a downstream target of AMPK (Fig. 7a, middle panel). In addition, the phosphorylation of p70 ribosomal protein S6 kinase (p70S6K) and eIF4E-binding proteins 1 (4E-BP1), two well-known mTOR substrates, were also markedly declined following treatment with AR-A (Fig. 7a, lower panel). We also found that there was a decrease in TFEB nuclear translocation after the AMPK inhibitor compound C (C.C) treatment in AR-A pretreated-HepG2 cells (Fig. 7b). Furthermore, a significant reduction in the siGSK-3β knockdown-mediated c-FLIP_L_ degradation was also observed in MHCC97H cells pretreated with C.C, similar to that in AR-A treatment (Fig. 7c). Moreover, the combination of C.C and AR-A markedly reduced AR-A-induced lysosomal protease activities in MHCC97H cells (Fig. 7d). Collectively, these results suggest that inhibition of GSK-3β induces TFEB nuclear translocation via the AMPK-mTOR signaling pathway.

## Discussion

Although GSK-3β is ubiquitously expressed, the levels of GSK-3β expression vary widely among the various types of cells and tumor tissues. The roles of GSK-3β in HCC remain controversial. Several studies have suggested possible roles of GSK-3β as a tumor suppressor gene in HCC, whereas, other studies have indicated that GSK-3β is a potential therapeutic target for this cancer. In this study, we demonstrated that GSK-3β is overexpressed and active in HCC cell lines compared to normal cells (Fig. 1). Pharmacological inhibition and genetic depletion of GSK-3β decrease the survival and induce caspase-dependent apoptosis in HCC cells (Fig. 2). Our results thus suggest GSK-3β perfect as a novel potential therapeutic target in the treatment of HCC.

Overexpression of c-FLIP_L_ has been observed in several types of cancer progression including colorectal carcinoma, pancreatic carcinoma, and HCC^37,38^. It has been shown that GSK-3β inhibition potentiates c-FLIP_L_ degradation in human lung cancer cells^24,39^. In our study, we could reproduce this biologic phenomenon in HCC cells (Fig. 2). We found that inhibition of GSK-3β with either siRNAs or small-molecule inhibitors was sufficient to attenuate c-FLIP_L_ levels in HCC. Complementarily, enforced expression of GSK-3β increased c-FLIP_L_ levels (Fig. 3). Thus, our findings clearly show that GSK-3β positively regulates c-FLIP_L_ levels in HCC, supporting the concept that GSK-3β acts as a tumor promoter by enhancing c-FLIP_L_ expression in HCC.

The role of GSK-3β in regulation of lysosome-dependent c-FLIP_L_ degradation is an important finding of this work. It is known that c-FLIP_L_ proteins are subjected to rapid turnover regulated through ubiquitin/proteasome-mediated protein degradation^25,40,41^. However, the signaling event that triggers c-FLIP_L_ degradation has not been characterized. In the current study, we found that the inhibition of GSK-3β with AR-A did not increase c-FLIP_L_ mRNA levels and that the presence of the proteasome inhibitor MG132 and lysosome inhibitor BAF prevented GSK-3β knockdown- or AR-A-induced c-FLIP_L_ downregulation, indicating GSK-3β inhibition-mediated c-FLIP_L_ degradation is mediated through both the proteasome-dependent and lysosome-dependent degradation pathway. Moreover, inhibition of GSK-3β induced biogenesis of functional lysosomes, promoted translocation of c-FLIP_L_ to lysosomes, indicating that c-FLIP_L_ could be degraded through the lysosomal pathway. Collectively, our findings reveal a novel mechanism by which inhibition of GSK-3β promotes lysosome-dependent degradation of c-FLIP_L_.

Lysosomes are cytoplasmic membrane-enclosed organelles containing hydrolytic enzymes that degrade macromolecules and cell components^42,43^. Lysosome dysfunction causes lysosome storage diseases, neurodegenerative disorders, and cancer cell invasion, while enhanced lysosome biogenesis promotes clearance of damaged organelles or aggregated proteins that can cause disease^44,45^. In our study, cell fractionation and confocal microscopy analysis indicated that c-FLIP_L_ was colocalized with LAMP1 (lysosome marker) and GSK-3β inhibition promoted translocation of c-FLIP_L_ to the lysosomes (Figs. 5 and 6). How c-FLIP_L_ was degraded in lysosomes is by far less clear. The simplest hypothesis is that the YVWL-containing signal peptide in c-FLIP_L_ directed it fused with prelysosomes or undergo acidization to form acid lysosomes and to be degraded. Whether other mechanisms involved in lysosomal degradation of c-FLIP_L_ requires further investigations.

How lysosomal biogenesis occurs is not completely understood. Recent study demonstrated that lysosome biogenesis can be triggered by the transcription factors TFEB^43^, which activated lysosomal biogenesis and promoted protein degradation^46^. Our study provides further support for a role of GSK-3β in the regulation of TFEB in HCC. A study in pancreatic cancer cells has shown that GSK-3β inhibition leads to nuclear translocation of TFEB^47^. Our data support such regulation in HCC cells. Inhibition of GSK-3β results in an increased number of lysosomes and higher levels of lysosomal enzymes (Fig. 4c–f), thus enhancing lysosomal catabolic activity for c-FLIP_L_ degradation. In addition, TFEB knockdown can reverse the effects of GSK-3β inhibition on c-FLIP_L_ levels (Fig. 6e). These findings suggest a new mechanism of GSK-3β regulating the level of c-FLIP_L_ through the TFEB-dependent lysosomal pathway.

Since mTOR participates in regulating TFEB activity^48^, we examined the potential roles for mTOR in GSK-3β-regulated TFEB activity. We found that GSK-3β is a positive regulator of mTOR (Fig. 7a). This pathway is known to promote cell proliferation and cancer development^49^. Inhibition of cell proliferation observed with GSK-3β inhibitors is likely due to their ability to inhibit mTOR, which mimics the anticancer activity of rapamycin^50,51^. Furthermore, we demonstrated that GSK-3β inhibition-induced suppression of mTOR act through AMPK activation. Our studies are in agreement with previous work that GSK-3β interacts with AMPK and inhibits AMPK activity, thus preventing the suppressive effects of AMPK on mTOR^34,52^. In contrast, results from other analyses indicate that GSK-3β inhibits mTOR. One study revealed that a coordinated phosphorylation of TSC2 by GSK-3β and AMPK suppresses mTOR activity^53^. Thus, the role of GSK-3β in regulation of mTOR may depend on cell type or cell context. This issue needs further clarification.

In summary, this study shows a novel mechanism by which inhibition of GSK-3β induces c-FLIP_L_ degradation through lysosome-dependent manner. Through this study, we are able to show, for the first time, that GSK-3β inhibition induced-TFEB translocation and subsequent biogenesis of functional lysosomes is associated with the induction of c-FLIP_L_ degradation, thus defines a novel cellular process induced by GSK-3β inhibitors in the HCC treatment.

## Materials and methods

### Reagents and antibodies

Antibodies against phospho-GS(Ser641), LC3, p62, AMPK, phospho-AMPK (Thr172), mTOR, phospho-mTOR(Ser2448), phospho-p70S6K (Thr389), p70S6K, phospho-4E-BP1(Thr37/46), 4E-BP1 were purchased from Cell Signaling (Danvers, MA, USA). Anti-GSK-3β and β-catenin antibodies were purchased from Santa Cruz (Santa Cruz, CA, USA). Antibodies against LAMP1, cathepsin B, TFEB, H3, and MG132, BAF were purchased from Sigma (St. Louis, MO, USA). Anti-FLIP antibody was purchased from ENZO Life sciences (Farmingdale, NY, USA). Human recombinant TRAIL was purchased from PeproTech (Rocky Hill, NJ, USA).GSK-3β inhibitors AR-A014418, SB216763, and SB415286 were obtained from Selleck (Shanghai, China).

### Plasmids and HCC specimens

Plasmid pCDNA3-HA-GSK-3β wild type, the constitutively inactive type (pCDNA3-HA-GSK-3β-K85A), and active mutants (pCDNA3-HA-GSK-3β-S9A) of GSK-3β were kindly provided by Dr. James Woodgett (Department of Medical Biophysics, University of Toronto, Canada). The pGL2-2xCLEAR-luciferase plasmid (#81120) and pEGFP-TFEB plasmid (# 38119) were purchased from Addgene (Cambridge, MA, USA). Tumorous liver tissues and the corresponding adjacent nontumoral liver tissues were obtained from six patients who underwent curative surgery for HCC at Beijing friendship hospital.

### Cell culture and transfection

HCC cell lines MHCC97H, HepG2, Hep3B, BEL7402, SMMC7721, Huh7, and human normal liver cell line HL7702 were purchased from Shanghai Institutes for Biological Sciences (Shanghai, China). All cells were cultured in Dulbecco’s modified Eagle’s medium (Gibico, NY, USA) supplemented with 10% fetal bovine serum (Hyclone, UT, USA), 100 U/mL penicillin, and100 μg/mL streptomycin sulfate and incubated in a humidified 5% CO_2_ atmosphere at 37 °C. Cells were transfected with Lipofectamine 2000 (Invitrogen, CA, USA) according to the manufacturer’s recommendations. For siRNA-mediated silencing, cells were transfected with 100 nM of siGSK-3β (#1, 5’-AGUUUGACAUUUGGGUCCC-3′; #2, 5’-UGUUUCCGGAACAUAGUCC- 3′) or siTFEB (5’-UGUAAUGCAUGACAGC CUG-3′) (GenePharma, Shanghai, China) siRNAs and a control siRNA. Forty-eight hour post-transfection, the protein expression was analyzed by IB. For the expression of TFEB-EGFP, cells were transfected for 48 h with the pEGFP-TFEB followed by 6 h treatment with DMSO or AR-A. Cells were either lysed for protein expression analysis by IB or processed for fluorescence microscopy.

### Measurement of cell viability and apoptosis

Cell viability was detected using CellTiter 96 AQueous One Solution Cell Proliferation Assay (MTS assay, Promega, Madison, WI, USA). Detection of apoptotic cells was performed using Annexin V-FITC/PI apoptosis detection kit (KeyGen, Nanjing, China) as described previously^54^.

### Cell impedance assay

The growth of GSK-3β-silenced cells was determined by cell impedance assay. Briefly, 50 μL of DMEM supplemented with 10% FBS was placed in each well of the E-plate 16 (ACEA Biosciences, San Diego, CA, USA). The final volume in a single well was adjusted to 100 μL of cell culture medium by adding additional 50 μL medium containing 1000 cells. Each treatment includes two replicates. The E-plates were incubated at room temperature for 30 min in a laminar flow cabinet and then placed on the RTCA DP Station (ACEA Biosciences) located in an incubator at 37 °C for continuous impedance recording. After 24 h incubation, the siRNAs targeting GSK-3β were put into the medium. Cell Index values measured by continuous impedance recordings every 15 min reflected the cell activities.

### Immunoblotting

IB was performed as described previously^54^. Briefly, cells were washed with ice-cold PBS and lysed in M2 lysis buffer (20 mM Tris-HCl, pH 7.5, 150 mM NaCl, 10 mM β-glycerophosphate, 5 mM EGTA, 1 mM sodium pyrophoshate, 5 mM NaF, 1 mM Na_3_VO_4_, 0.5% Triton X-100, and 1 mM DTT) supplemented with protease inhibitor cocktail (Sigma, P8340). Proteins were separated by SDS-PAGE and electrically transferred to a polyvinylidene difluoride membrane. The membrane was probed with the appropriate primary antibody and with a HRP-conjugated secondary antibody. Blots were visualized by Tanon 5200 system (Tanon, Shanghai, China).

### Nuclear and cytoplasmic fractionation

Nuclear and cytoplasmic fractions were extracted from cell homogenates by Nuclear/Cytosol Fractionation Kit (K266-25, BioVision, Milpitas, CA, USA) according to the manufacturer’s protocol. Briefly, 2 × 10^6^ cells were collected by centrifugation for 10 min at 600 g at 4 °C. Cell pellet were resuspend in 0.2 mL CEB-A Mix buffer, followed by adding 11 μL of ice-cold Cytosol Extraction Buffer-B and centrifuging for 5 min at 16,000 g, the supernatant (cytoplasmic extract) was transferred to a clean pre-chilled tube, and the pellet (contains nuclei) was resuspend in 100 μL of ice-cold Nuclear Extraction Buffer Mix. The nuclear mix was centrifuge at 16,000 g for 10 min, and supernatant (nuclear extract) was transfer to a clean pre-chilled tube. The nuclear and cytosol fractionations were subjected to IB.

### LysoTracker red staining

Lysosomes were labeled by incubating cells with the LysoTracker Red DND-99 dye (50 nM) (Invitrogen, L-7528) for 30 min at 37 °C. The medium was aspirated and washed twice quickly in PBS to remove unbound LysoTracker, and then recorded the fluorescence by microscope (Zeiss, Axio Vert.A1).

### Quantitative real-time PCR (qRT-PCR)

Total messenger RNA from HepG2 or MHCC97H cells was isolated by Trizol reagent (Invitrogen). First-strand cDNA synthesis and PCR reaction were conducted as described before^55^. Total RNA was normalized in each reaction using GAPDH cDNA as an internal standard. The primer of target genes were as following: *Atp6v1h* (sense 5′-GGAAGTGTCAGATGATCCC A-3′, anti-sense 5′-CCGTTTGCC TCGTGGATAAT-3′); *Tfeb* (sense 5′-CCATCCC CATTCCATCACCT-3′, anti-sense 5′-ACAGAAGTGGATCAGAGGCC-3′); *Ctsb* (sense 5′-AGTGGAGAATGGCACACCCTA-3′, anti-sense 5′-AAGAAGCCATTGT CACCCCA-3′); *Vps11* (sense 5′-ATACCACCCTGCTCCTCAAC-3′, anti-sense 5′-CAGATACAGGGCATGGGAG T-3′); *Map1lc3b* (sense 5′-CGCACCTTCGAAC AAAGAGT-3′, anti-sense 5′-AGCT GCTTCTCACCCTTGTA-3′); *Ctsf* (sense 5′-A CAGAGGAGGAGTTCCGCACTA -3′, anti-sense 5′-GCTTGCTTCATCTTGTTGC CA-3′); *Atg9b* (sense 5′-ACCTCCTCCTCCTCCTTCAT-3′, anti-sense 5′- GTGGGA GGGGAAAATGAGGA-3′); *Lamp1* (sense 5′-ACGTTACAGCGTCCAGCTCAT-3′, anti- sense 5′-TCTTTGGAG CTCGCATTGG-3′); *Gapdh* (sense 5′-TGCACCACCA ACTGCTTAGC-3′, anti-sense 5′-GGCATGGACTGTGGTCATGAG-3′).

### NAG assay

NAG assays were performed using a kit from Sigma (CS0780), based on the principle that NAG hydrolyses 4-nitrophenyl N-acetyl-β-D-glucosaminide (NP-GlcNAc) to generate p-nitrophenol that can be measured colorimetrically at 405 nm following ionization at basic pH. Briefly, cells treated with AR-A (20 μM) for 3 h were lysed in RIPA buffer (250 μL). Ten micrograms of cell lysates from each sample were normalized to equal volume and measured in triplicate for NAG activity following the protocol provided by the supplier.

### Confocal microscopy

Cells were seeded at 70% confluence on Lab-Tek chamber slide and were transfected with the indicated plasmids at the second day. After 24 h, the cells were treated with vehicle (DMSO) or AR-A (20 µM). Following incubations, the cells were washed with PBS, fixed with 4% (wt/vol) paraformaldehyde for 30 min at room temperature. The cells were then examined with confocal microscopy (LSM710, Zeiss, Germany). Quantification of colocalization of the two labels (green and red) was conducted using the ‘Colocalization’ module of Image Pro plus 6.0.

### Statistics

Data were statistically analyzed with GraphPad Prism 5 by using either one-way analysis of variance (ANOVA) (followed by either a Dunnett’s test when referring to the control only or Tukey’s post hoc test otherwise) or the Student *t* test.

## Electronic supplementary material

Supplementary Information

Supplementary Figure S1

Supplementary Figure S2

Supplementary Figure S3

Supplementary Figure S4
