# Supplementary material for: Glycogen synthase kinase-3β inhibition promotes lysosome-dependent degradation of c-FLIPL in hepatocellular carcinoma
Source: Cell Death Dis. 2018 Feb 14;9(2):230. doi: 10.1038/s41419-018-0309-3 (PMC5833564; doi:10.1038/s41419-018-0309-3)
Supplement: Supplementary file 1 — Supplementary Information [file 41419_2018_309_MOESM1_ESM.docx]

**Supplementary Information**

**Glycogen synthase kinase-3β inhibition promotes lysosome-dependent degradation of c-FLIP_L_ in hepatocellular carcinoma**

Na Zhang^†1^, Xiaojia Liu ^†1^, Lu Liu ^1^, Zhesong Deng^2^, Qingxuan Zeng^1^, Weiqiang Pang^1^, Yang Liu^1^, Danqing Song^*1^, Hongbin Deng ^*1^

**Materials and Methods**

**Plasmid and antibody.** pCMV3-N-myc-FLIP_L_ plasmid was purchased from Sino Biological Inc (Beijing, China). Anti-myc antibody was purchased from Cell Signaling (Danvers, MA, USA).

**Measurement of cell viability.** Cell viability was detected using a Lactate dehydrogenase (LDH) release assay according to the manufacturer’s guidelines of CytoTox 96 Non-Radioactive Cytotoxicity Assay (Promega, Madison, WI, USA).

**Hoechst 33342 staining assay.** HepG2 cells treated with AR-A or plus TRAIL for 24 h were stained with Hoechst 33342 (1 μg/ml, sigma) in the dark at 37 °C for 10 min. After being washed with phosphate-buffered saline (PBS), the cells were observed using a fluorescence microscope (Carl Zeiss, Göttingen, Germany).

**Supplementary Figure Legends**

**Supplementary Figure S1.** HL7702, BEL7402, Hep3B, SMMC7721, HepG2 and MHCC97H cells were planted in 6-well plates, the number of formed clones were counted. Data are presented as means ± S.D. (*n*=3). ^*^*P* < 0.05, ^**^*P*<0.01, ^***^*P*<0.001 compared with the HL7702 group.

**Supplementary Figure S2.** (**a**) HepG2 and MHCC97H cells were treatment with AR-A at the indicated concentrations for 24 h, the expression level of p-GS was determined by IB. (**b**) HepG2 and MHCC97H cells were treated with AR-A at the indicated concentrations, cell viability was measured by LDH release assay. Data are presented as means ± S.D. (*n*=3). ^*^*P* < 0.05,^**^*P*<0.01 compared with DMSO group.

**Supplementary Figure S3.** (**a**) HepG2 cells were treatment with 20 μM AR-A in the presence or absence of Z-VAD-FMK (20 μM) for 24 h, the apoptotic cell death was determined by Annexin V/PI staining. (**b**) HepG2 cells treated with 20 ng/mL TRAIL alone, 20 μM AR-A alone, or their combinations were stained with Hoechst 33342 (1 μg/mL) and then observed under a fluorescence microscope. Apoptotic cells with chromatin shrinking were observed (as indicated by the arrow). (**c**) MHCC97H cells were treated with 20 μM AR-A alone, 20 ng/mL TRAIL alone, or their respective combinations in the presence or absence of necrostain-1(40 μΜ). After 24 h, the cell viability was determined by LDH release assay. (*n*=3). ^*^*P*<0.05 compared with TRAIL group.

**Supplementary Figure S4.** HepG2 cells were transfected with empty vector or expression plasmids carrying myc-c-FLIP_L_ for 36 h, followed by DMSO or 20 μM AR-A treatment for 24h.The expression levels of myc-c-FLIP_L_ and β-actin were measured by IB (**a**). The apoptosis was measured by Annexin V/PI staining (**b**).
