# Supplementary figures and images for: Glycogen synthase kinase-3β inhibition promotes lysosome-dependent degradation of c-FLIPL in hepatocellular carcinoma
Source: Cell Death Dis. 2018 Feb 14;9(2):230. doi: 10.1038/s41419-018-0309-3 (PMC5833564; doi:10.1038/s41419-018-0309-3)

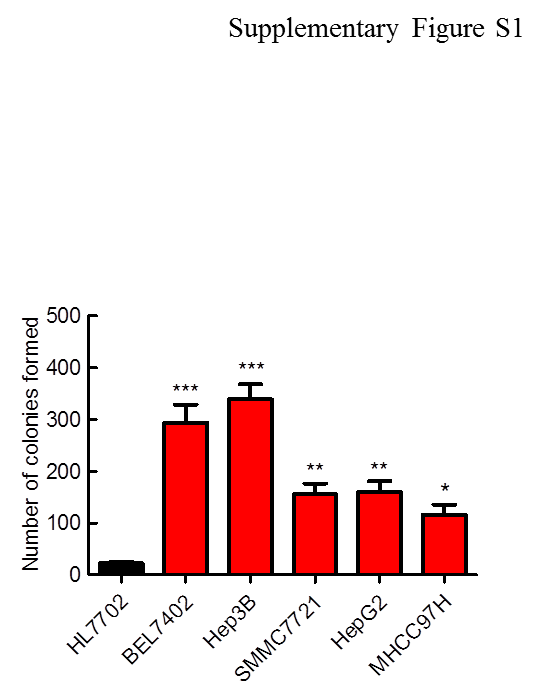

Supplement: Supplementary file 2 — Supplementary Figure S1 [file 41419_2018_309_MOESM2_ESM.tif]

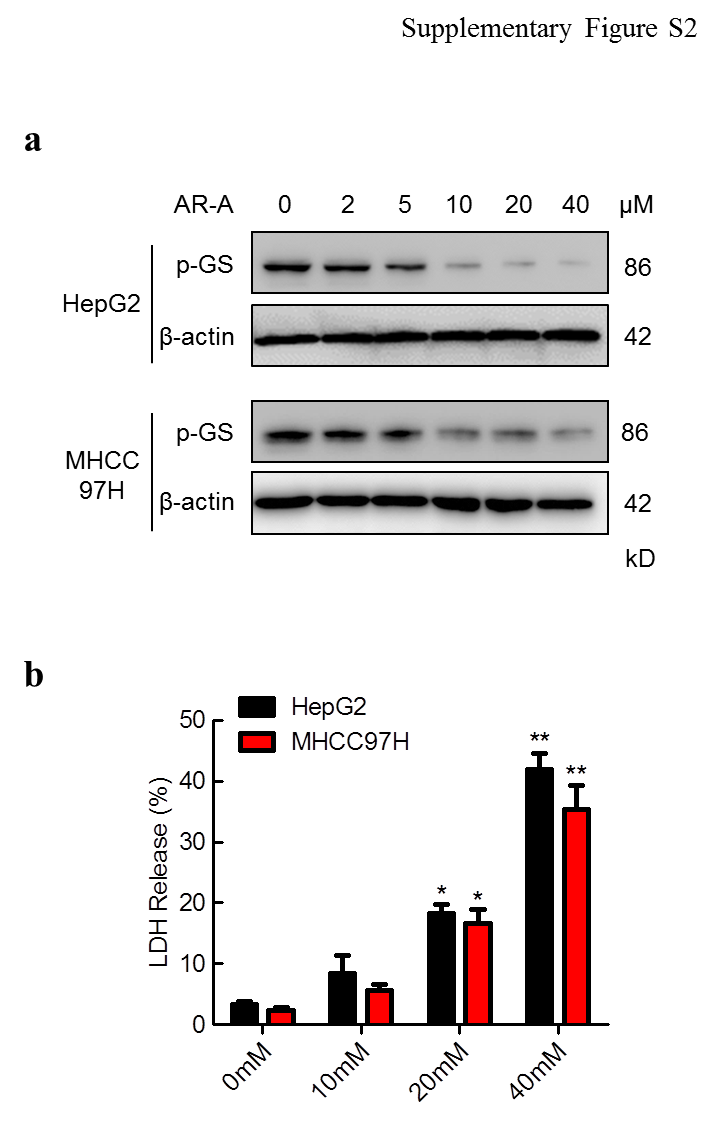

Supplement: Supplementary file 3 — Supplementary Figure S2 [file 41419_2018_309_MOESM3_ESM.tif]

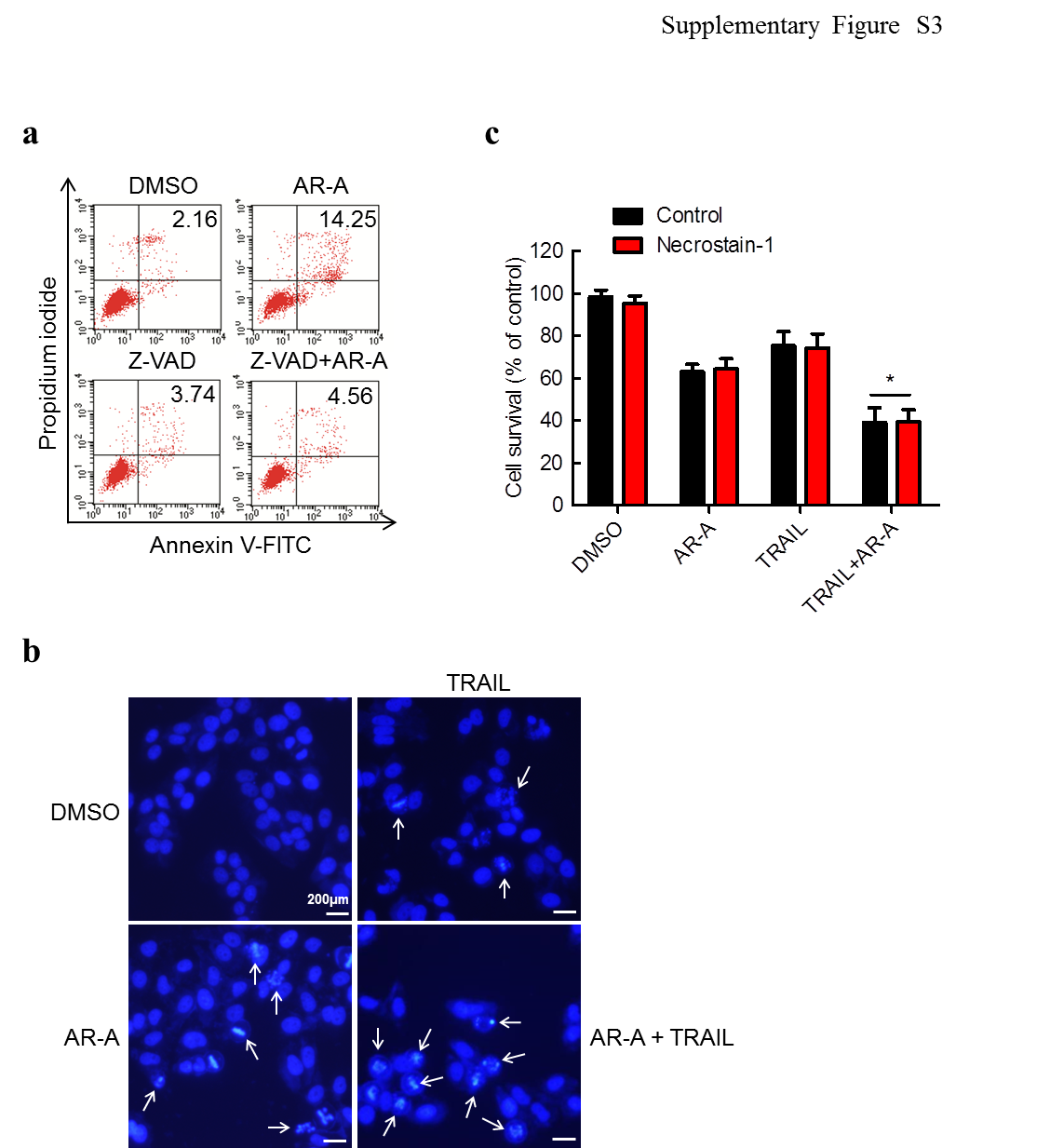

Supplement: Supplementary file 4 — Supplementary Figure S3 [file 41419_2018_309_MOESM4_ESM.tif]

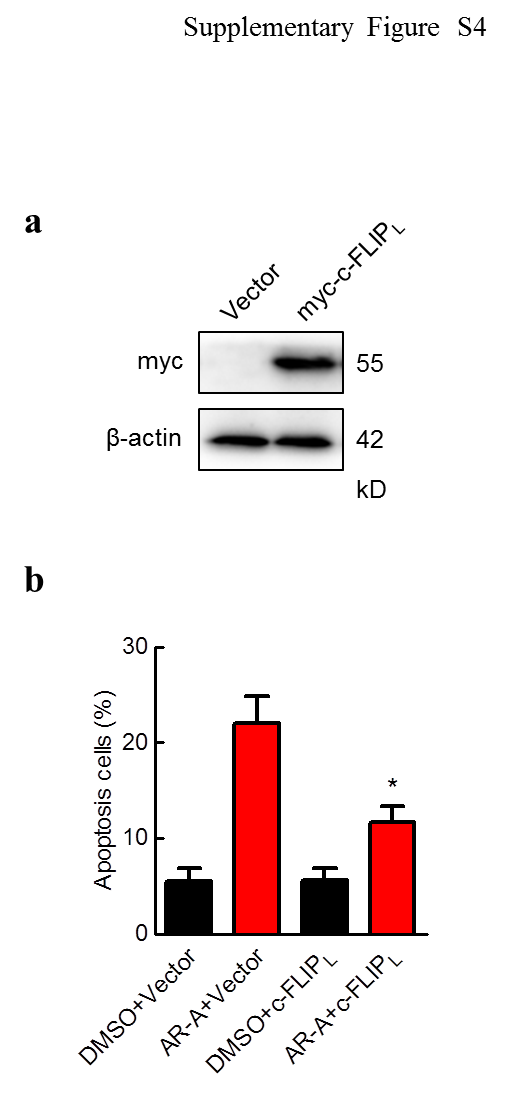

Supplement: Supplementary file 5 — Supplementary Figure S4 [file 41419_2018_309_MOESM5_ESM.tif]
